# Antibodies binding diverse pertactin epitopes protect mice from *Bordetella pertussis* infection

**DOI:** 10.1016/j.jbc.2022.101715

**Published:** 2022-02-11

**Authors:** Rui P. Silva, Andrea M. DiVenere, Dzifa Amengor, Jennifer A. Maynard

**Affiliations:** 1 Department of Molecular Biosciences, University of Texas, Austin, Texas, USA; 2 Department of Chemical Engineering, University of Texas, Austin, Texas, USA; 3 LaMontagne Center for Infectious Diseases, University of Texas, Austin, Texas, USA

**Keywords:** *Bordetella*, acellular vaccine, bactericidal antibodies, virulence factors, phage display, whooping cough, ACT, adenylate cyclase toxin, BG, background, BLI, biolayer interferometry, BSA, bovine serum albumin, cfu, colony-forming unit, Fha, filamentous hemagglutinin, HBSS, Hank's balanced salt solution, HRP, horseradish peroxidase, IgG, immunoglobulin G, mAb, monoclonal antibody, PBS-T, PBS with Tween-20, RGD, Arg–Gly–Asp, scFv, single-chain variable fragment, SSM, Stainer–Scholte media

## Abstract

Infection by the bacterium *Bordetella pertussis* continues to cause considerable morbidity and mortality worldwide. Many current acellular pertussis vaccines include the antigen pertactin, which has presumptive adhesive and immunomodulatory activities, but is rapidly lost from clinical isolates after the introduction of these vaccines. To better understand the contributions of pertactin antibodies to protection and pertactin's role in pathogenesis, we isolated and characterized recombinant antibodies binding four distinct epitopes on pertactin. We demonstrate that four of these antibodies bind epitopes that are conserved across all three classical *Bordetella* strains, and competition assays further showed that antibodies binding these epitopes are also elicited by *B. pertussis* infection of baboons. Surprisingly, we found that representative antibodies binding each epitope protected mice against experimental *B. pertussis* infection. A cocktail of antibodies from each epitope group protected mice against a subsequent lethal dose of *B. pertussis* and greatly reduced lung colonization levels after sublethal challenge. Each antibody reduced *B. pertussis* lung colonization levels up to 100-fold when administered individually, which was significantly reduced when antibody effector functions were impaired, with no antibody mediating antibody-dependent complement-induced lysis. These data suggest that antibodies binding multiple pertactin epitopes protect primarily by the same bactericidal mechanism, which overshadows contributions from blockade of other pertactin functions. These antibodies expand the available tools to further dissect pertactin's role in infection and understand the impact of antipertactin antibodies on bacterial fitness.

Whooping cough is a highly contagious respiratory disease caused primarily by the gram-negative bacterium *Bordetella pertussis*, although *Bordetella bronchiseptica* and *Bordetella parapertussis* can also cause human disease. Current acellular pertussis vaccines introduced in the mid-1990s comprise pertussis toxin, filamentous hemagglutinin (Fha), and pertactin in the common trivalent vaccines with fimbriae 2/3 also included in pentavalent formulations. While there is no established serological correlate of protection (1), antipertussis toxin and anti-Fha titers correlate with susceptibility to disease (2), but only pertactin induces bactericidal antibodies that mediate bacterial clearance (3, 4). Accordingly, pertactin-containing vaccines were more effective than those lacking pertactin in vaccine efficacy trials (5). In mouse experiments, pertactin vaccination induces opsonic and bactericidal sera that reduce respiratory tract colonization after experimental infection with *B. pertussis* (6, 7).

Unlike pertussis toxin and Fha, mature pertactin is only found associated with the bacterial surface, providing a target for bactericidal antibodies. Pertactin is expressed as a 93 kDa precursor protein, including an N-terminal secretion signal, a 60 kDa central passenger domain, and a 30 kDa C-terminal porin domain (Fig. 1*A*). After translocation and cleavage from the porin domain, the passenger domain remains noncovalently bound to the outer membrane. This domain is a 16-stranded parallel right-handed beta helix with individual rungs connected by loops of varying lengths (8), two of which are particularly large and variable proline-rich repeat regions named R1 and R2 (Fig. 1*B*). The 13 observed *B. pertussis* pertactin alleles have varying numbers of these repeat sequences (9), as does pertactin produced by the related strains *B. parapertussis* and *B. bronchiseptica* (10), with additional residue changes outside the repeat regions (93.4 and 93.6% identity to *B. pertussis* pertactin, respectively).

**Figure 1:**
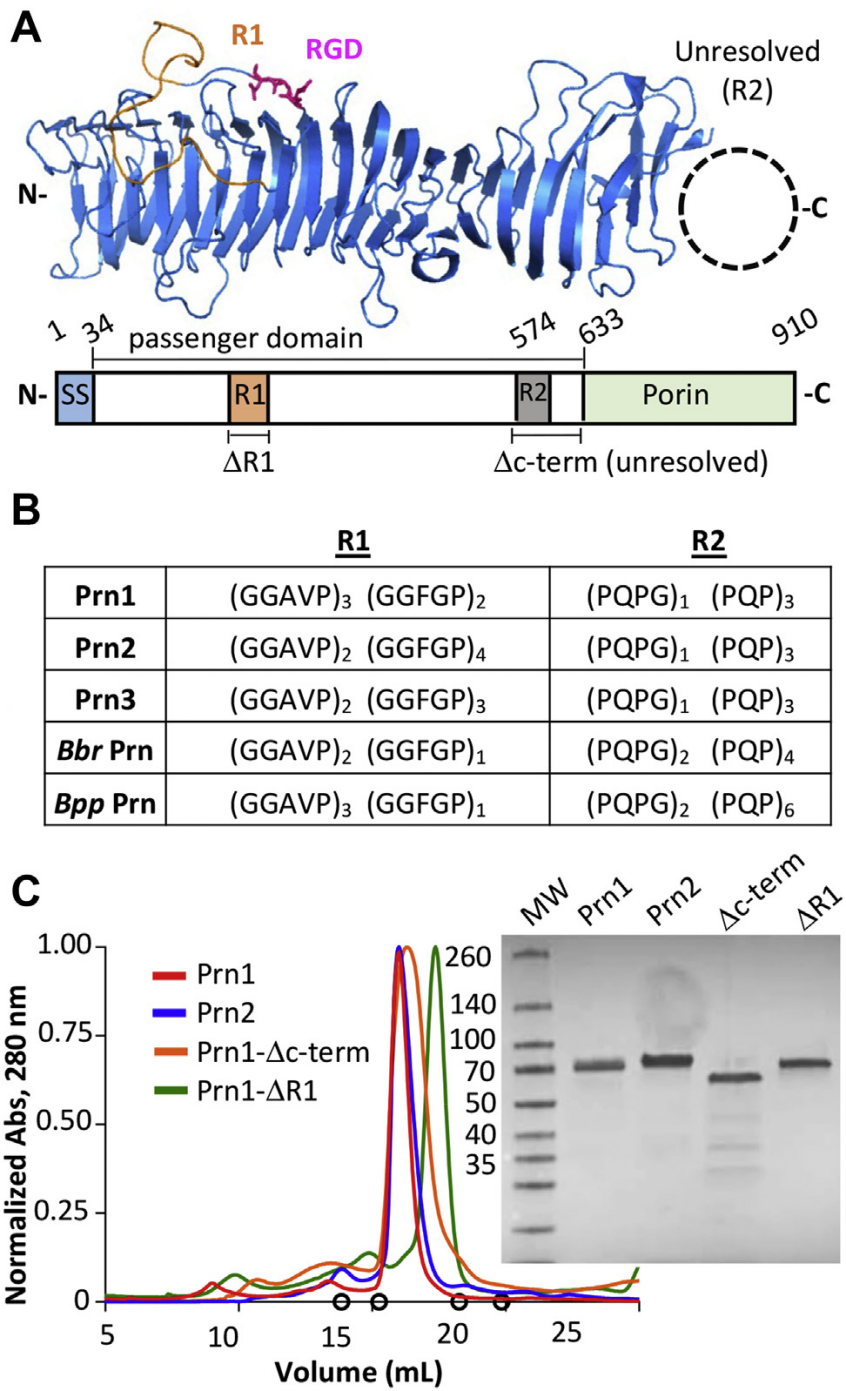
**Production of recombinant Prn1 and Prn2.***A*, pertactin passenger domain structure is shown with loop R1 in *orange* and the RGD in *purple*; residues 574 to 633 are unresolved (Protein Data Bank: 1DAB). Variants generated include Prn1-ΔR1 pertactin with residues 231 to 256 of the passenger domain replaced by (G_4_S)_2_, whereas Prn1-Δc-term truncated the structurally unresolved region after residue 633. *B*, sequence variation of the R1 and R2 loops shown for *Bordetella pertussis* Prn1, Prn2, and Prn3 and *B. bronchiseptica* (*Bbr*) and *B. parapertussis* (*Bpp*). Outside these regions, *B. pertussis* alleles are identical and share 93.6% and 93.4% amino acid identity to *Bbr* and *Bpp* pertactin, respectively; *Bbr* and *Bpp* pertactin are 99.5% identical outside of R1 and R2. *C*, recombinant and refolded pertactin was purified by size-exclusion chromatography with an S200 column on an Åkta FPLC with molecular weight assess by SDS-PAGE gel. FPLC standards are shown in *circles*: cytochrome C (12.4 kDa, 19.85 ml), carbonic anhydrase (29.0 kDa, 18.26 ml), ovalbumin (44 kDa, 16.68 ml), albumin (66.0 kDa, 15.26 ml), and aldolase (158 kDa, 13.85 ml). RGD, Arg–Gly–Asp.

The pertactin genes in clinical pertussis isolates have changed dramatically in recent decades. The Prn2 and Prn3 alleles with varying R1 sequences emerged and became dominant over Prn1 after the introduction of Prn1-containing whole-cell vaccines (11). Similar sequence changes were observed in *B. bronchiseptica* strains recovered from vaccinated pigs (12). After the introduction of pertactin-containing acellular vaccines, pertactin-deficient isolates became increasingly prevalent because of a variety of mutations (13, 14). Interestingly, pertactin-deficient strains are only slightly attenuated in naive individuals but have increased fitness in vaccinated individuals (9), raising questions about pertactin's role in infection. Pertactin was initially thought to mediate bacterial adhesion to eukaryotic cells *via* an Arg–Gly–Asp (RGD) motif (15, 16), but this role remains uncertain and no receptor has yet been identified. More recently, pertactin immunomodulatory activities were described, but no binding partners have been identified (17).

Antibodies can be useful tools to dissect the functions of virulence factors, but few antibodies binding pertactin have been characterized. These include several antibodies binding linear epitopes in the R1 and R2 loops (18, 19), but it is unknown whether these antibodies represent the range of those induced by acellular pertussis vaccination or infection. One of these, PeM4 binding the R1 loop, protected mice from *B. pertussis* infection after passive administration (18). Bacterial surface antigens from other bacteria share key characteristics with pertactin, including the *Streptococcus pyogenes* M protein, which also includes a hypervariable region. Interestingly, only antisera binding the hypervariable region, but not two other surface-exposed regions, of the representative M5 variant, are opsonic (20). Similarly, a monoclonal antibody (mAb) binding the *Streptococcus pneumoniae* histidine triad protein near the membrane anchor point better bound whole bacteria and better protected mice against lethal infection than an antibody binding the more surface-exposed C terminus (21).

Accordingly, we speculated that identification of antibodies binding different pertactin epitopes would help define pertactin's role in pathogenesis, structure–function relationships, and contributions to protection. We discovered and characterized a panel of recombinant antibodies binding pertactin from mice immunized with Prn1, which is included in human acellular vaccines. This defined four competition groups, which are conserved across *B. pertussis* alleles and appear immunogenic during *B. pertussis* infection. Surprisingly, antibodies representative of each competition group appeared functionally similar when expressed as recombinant mouse immunoglobulin G2a (IgG2a) isotypes. While all bound pertactin-expressing *B. pertussis* bacteria and mediated protection against bacterial challenge in mice that depended strongly on Fc effector functions, none mediated antibody-dependent complement lysis *in vitro*. Overall, these data suggest that antipertactin bactericidal immune responses overshadow other potential protective mechanisms such as blockade of pertactin–receptor or pertactin–immune interactions *in vivo*.

## Results

### Discovery of antibodies binding pertactin by phage display

The mature forms of Prn1 and Prn2 (residues 35–640 and 35–645, respectively) were expressed in *Escherichia coli* as inclusion bodies, solubilized, and purified by sequential streptactin and size-exclusion chromatographic steps (19, 22). Since the R1 and R2 loops were previously reported as being immunodominant, we also generated variants removing these loops: Prn1-ΔR1 with R1 replaced by a (Gly_4_Ser)_2_ glycine–serine linker and Prn1-Δc-term, with the C-terminal 67 residues including R2 truncated. Preparative size-exclusion chromatography showed a single peak corresponding to the expected ∼60.3 kDa molecular weight of mature pertactin, whereas SDS-PAGE gel showed a single band ∼70 kDa characteristic of intact pertactin for Prn1, Prn2, and Prn1-ΔR1, with Prn1-Δc-term appearing somewhat smaller, as expected (Fig. 1*C*).

Mice were immunized and boosted with Prn1, the same allele in human acellular vaccines, resulting in antipertactin titers of >1:10,000. These data suggested that the mice generated strong antibody responses to pertactin and would serve as a rich source of recombinant antibodies. Splenic mRNA of immunized mice was purified, reversed transcribed, and antibody-variable regions amplified as described (23). The heavy and light variable regions were combined to form single-chain variable fragments (scFv) and cloned en masse yielding a library of ∼2.2 × 10^8^ colonies. This was subjected to four rounds of panning with decreasing concentrations of pertactin. Screening identified 12 clones with variable gene usage and CDR3 sequences, especially in the heavy chain (Fig. S1), which exhibited strong binding to pertactin. These variable regions were expressed and purified separately with mouse IgG2a/kappa and human IgG1/kappa constant domains in ExpiCHO cells and purified by protein A chromatography (Fig. S2).

**Figure 2:**
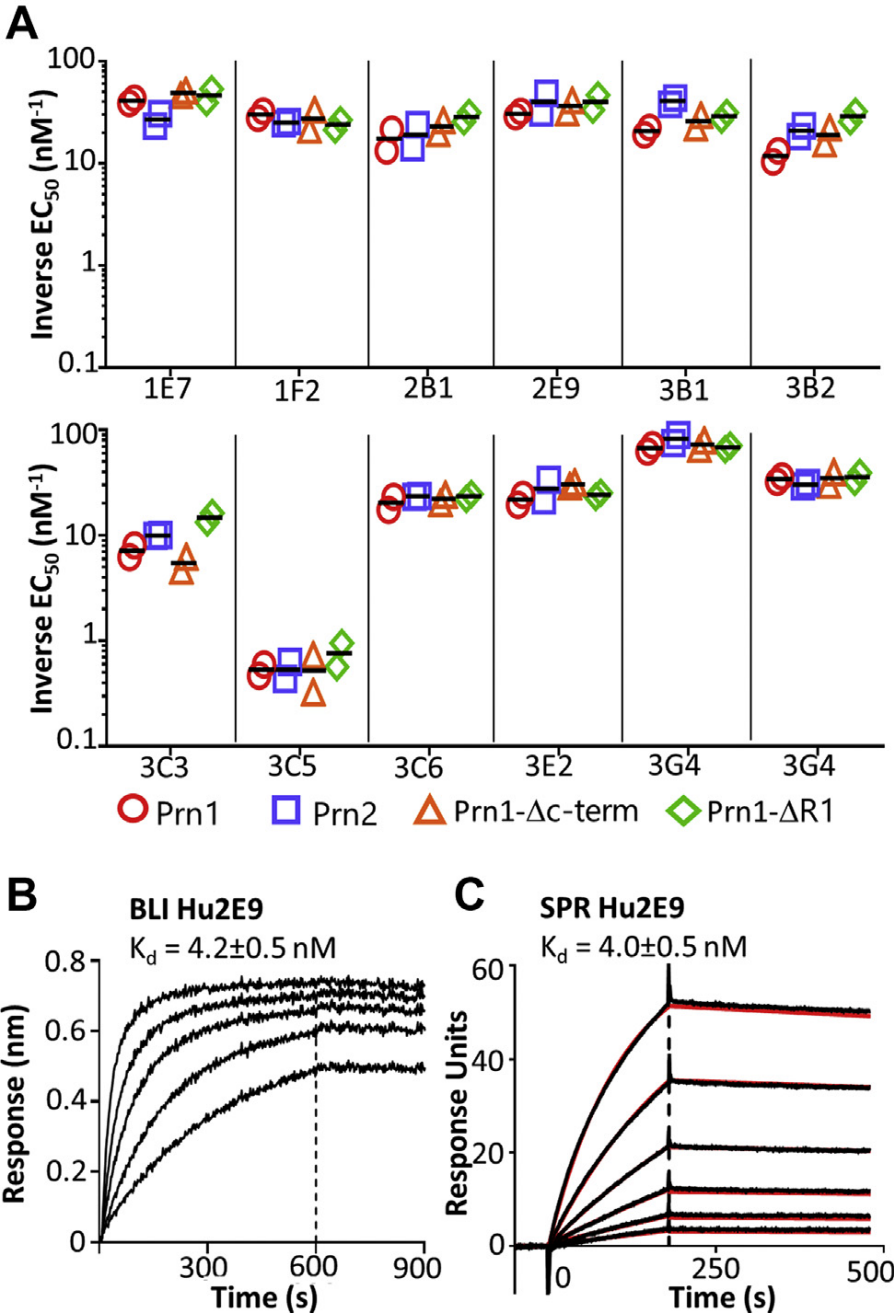
**Antibodies bind conserved Prn1 and Prn2 epitopes.***A*, binding of purified antibodies was compared by ELISA using coated pertactin, followed by serially diluted antibody in duplicate and detection with anti-huFc-HRP. EC_50_ values were determined from 4PL fits in GraphPad software, and inverse EC_50_ values were compared. *Symbols* depict replicate experimental results with means shown as a *line*. *B*, antibody 2E9 affinity was measured by BLI using antihuman Fc sensors coated with 2E9 and then dipped into wells with serial twofold pertactin dilutions from 100 to 6.25 nM. The apparent equilibrium *K*_*d*_ value was determined using a 1:1 Langmuir binding model with Octet analysis software. *C*, the kinetic *K*_*d*_ was determined by SPR using 2E9 immobilized on a CM5 chip to a total of 600 response units. Pertactin was injected at serial twofold dilutions from 250 to 7.8 nM. Kinetic on-rate and off-rate analyses were performed using BIAEvaluation 3.0 software and fit using the 1:1 binding model, with kinetic *K*_*d*_ = *k*_off_/*k*_on_. *Vertical dashed lines* indicate the beginning of the dissociation phase; mean and error from replicate experiments are shown. BLI, biolayer interferometry; HRP, horseradish peroxidase; SPR, surface plasmon resonance.

Initial characterization by ELISA showed that the antibody panel exhibited similar binding to all four Prn variants (Prn1, Prn2, Prn1-Δc-term, and Prn1-ΔR1), with EC_50_ values ranging from 0.1 to 0.01 nM, except clone 3C5, which bound more weakly (Fig. 2*A*). These data suggest that the antibodies bind conserved epitopes in Prn1 and Prn2 that are independent of the R1 and R2 regions. As a more quantitative method to rank antibodies by apparent Prn1-binding affinity, we used biolayer interferometry (BLI) with equilibrium analysis. Four antibodies exhibited apparent *K*_*d*_ values <10 nM, as is typical after *in vivo* selection (24); seven had modest affinities of 10 to 30 nM; with clone 3C5 having a weak >100 nM affinity (Fig. S3 and Table 1). To validate these BLI data, antibody 2E9 was subjected to kinetic analysis using surface plasmon resonance resulting in *k*_on_ of 3.6 × 10^4^ M^−1^s^−1^ and *k*_off_ of 1.4 × 10^−4^ s^−1^ and a calculated *K*_*d*_ of 3.9 ± 0.5 nM (Fig. 2, *B* and *C*). Since antibody affinity often correlates strongly with protective functions (25), it is helpful to compare antibodies that have similar *K*_*d*_ values, and this information was used to select antibodies for further characterization.

**Table 1:** Characteristics of antibody binding to pertactin^a^

Abbreviations: *Bbr*, *B. bronchiseptica* RB50; *Bpp*, *B. parapertussis* 12822; ND, not determined.

Number of plus-signs and gray-shaded area provide a qualitative comparison of the mean fluorescence intensity of Alexa Fluor-647 assessed by flow cytometry.

a *K*_*d*_ values represent the apparent equilibrium affinity for immobilized human IgG1 antibodies on antihuman IgG Fc tips, as measured by BLI with standard error shown. All fits had *R*^2^ > 0.95. To calculate effective *K*_*d*_ values, the geometric mean of the goat–antihuman Fc-Alexa Fluor 647 was fit using one-site specific Langmuir binding equation (*Y* = *B*_max_ ∗ *X*/(*K*_*d*_ + *X*). Affinity (*K*_*d*_), mean, and standard error for two experiments are reported.

### Antibodies bind conserved conformational epitopes accessible on cell-associated pertactin

**Figure 3:**
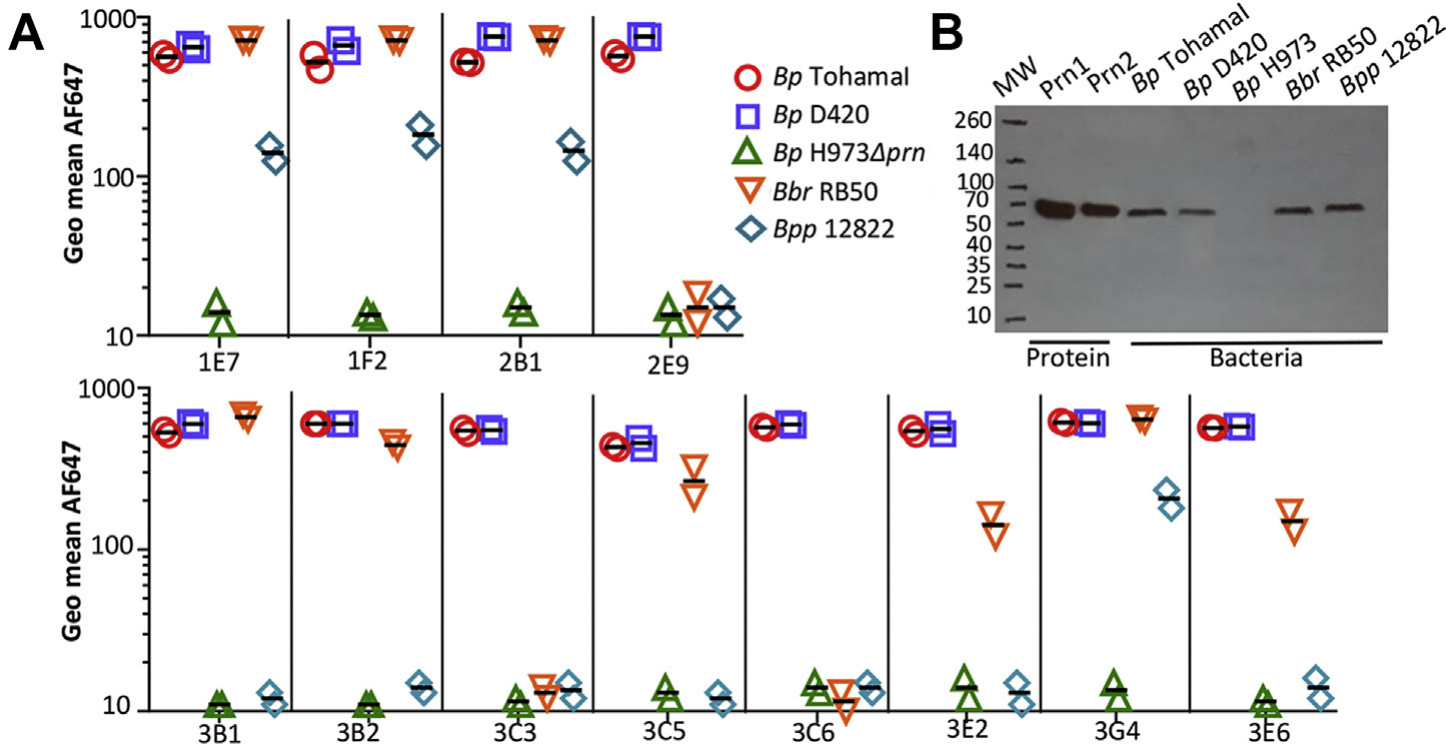
**Antibodies bind pertactin on *Bordetella* cells.***A*, to assess antibody binding to multiple *Bordetella* strains, midlog phase bacteria were incubated with human IgG1/κ antibodies at 20 μg/ml, followed by antihuman Fc-AF647 and analysis on a Fortessa flow cytometer. *B*, antibody 3G4 was used in a Western blot to detect midlog phase bacteria (10 μl of absorbance at 600 nm = 4 per lane) lysed and loaded onto a 4 to 20% gradient SDS-PAGE gel before transfer to a PVDF blot, with detection by anti-huFc-HRP. For reference, 100 ng purified Prn1 and Prn2 are also shown. *Bbr*, *B. bronchiseptica*; *Bp*, *B. pertussis*; *Bpp*, *B. parapertussis*; HRP, horseradish peroxidase; IgG, immunoglobulin G; PVDF, polyvinylidene difluoride.

Since pertactin remains associated with the bacterial surface, epitopes likely need to be accessible on intact bacteria to be relevant during infection. To determine which antibodies bind cell associated–pertactin and the breadth of pertactin homologs recognized, we performed flow cytometry with exponential bacteria, grown under Bvg+ conditions (Fig. S4). Similar to our ELISA data, all antibodies bind *B. pertussis* strains expressing Prn1 (TohamaI) and Prn2 (D420). No antibody bound the pertactin-deficient strain H973, a clinical isolate from the 2012 pertussis outbreak in Washington state, USA (26), confirming that the antibodies bind pertactin and have no affinity for other cell surface molecules. Five antibodies also bound pertactin expressed by *B. bronchiseptica* RB50, whereas four bound pertactin expressed by *B. parapertussis* 12822 (27), with antibodies 2B1, 1F2, 1E7, and 3G4 binding all three homologs, albeit with somewhat weaker affinity for *B. parapertussis* pertactin (Fig. 3*A* and Table 1). Since none of the epitopes involve the variable R1 and R2 repeats (Figs. 1 and 2), single amino acid polymorphisms appear to be responsible for these binding differences: outside of R1 and R2, Prn1 shares 93.6% amino acid similarity with *B. bronchiseptica* and 93.4% amino acid similarity with *B. parapertussis* pertactin.

Since previously reported antipertactin antibodies recognized epitopes with strong linear components (18, 19), we used Western blot analysis to determine which of the current antibody panel bind linear *versus* conformational epitopes. Only antibody 3G4 detected pertactin on a Western blot, suggesting its epitope includes a linear component (Fig. S5). This antibody bound pertactin from all strains by flow cytometry and also in Western blot (Fig. 3*B*). Overall, these antibodies recognize conformational pertactin epitopes that are accessible on the bacterial surface and conserved in *B. pertussis* alleles, with some also conserved across the three major *Bordetella* species. Accordingly, they may be relevant during infection with a range of *Bordetella* strains.

### Antibodies define four distinct competition groups on pertactin

**Figure 4:**
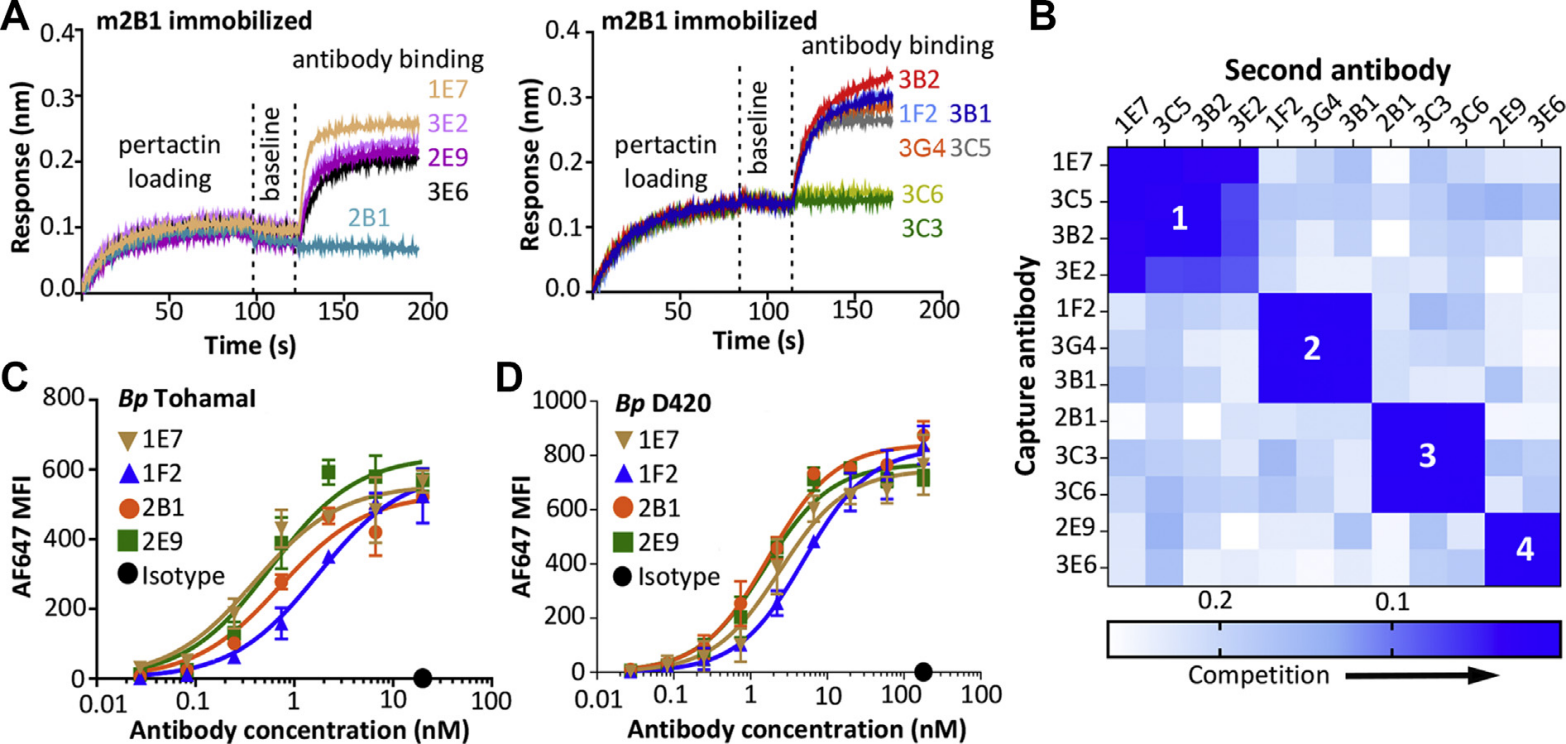
**Antibodies define four competition groups.** BLI competitive binding experiments used with antimouse IgG Fc sensors were loaded with a first antibody with mouse constant domains to 0.3 nm, then with Prn1 to reach baseline, then dipped into well with 100 nM of a second antibody with human constant domains. *A*, representative competition curves with 2B1-mIgG2a immobilized on the sensors and binding to all other antibodies. *B*, data for competition between all pairwise combinations of antibodies, summarized as a heat map using the signal obtained from the binding of the second antibody. *Darker blue* depicts lower binding of the second antibody and more competition; *lighter blue* depicts higher signal from second antibody and less competition. To determine the effective *K*_*d*_ for antibody binding to (*C*) *Bordetella pertussis* TohamaI and (*D*) D420 cells, antibodies (seven 3-fold serial dilutions from 27 to 0.01 μg/ml) were incubated with log-phase bacteria and analyzed by a Fortessa flow cytometer. Mean and standard deviations are shown from two independent experiments. BLI, biolayer interferometry; IgG, immunoglobulin G.

To determine which antibodies bind spatially distinct pertactin epitopes, we used a BLI competition assay. Pertactin was captured by an immobilized antibody and then allowed to bind a second antipertactin antibody. When antibody 2B1 was used to capture pertactin, all antibodies except 2B1, 3C3, and 3C6 bound the 2B1–peractin complex, indicating that these three antibodies define a competition group (Fig. 4*A*). We are only showing data for immobilized 2B1, but all possible pairwise comparisons were analyzed in this manner to define four competition groups, with data summarized in Figure 4*B*. As noted previously, some antibodies within a single competition group exhibit differential binding to pertactin homologs. Competition groups are defined by overlapping epitopes that sterically inhibit the simultaneous binding of two antibodies, but competing antibodies can engage different pertactin residues within that epitope. Thus, 3C3 and 2B1 can be in the same competition group, but amino acid polymorphisms in *B. bronchiseptica* pertactin could affect binding to antibody 3C3 but not antibody 2B1, as we observed (Table 1).

Since antibodies binding different epitopes often have grossly different functions, we selected a representative antibody from each competition group with the highest apparent affinity for further analysis: three binding pertactin from all classical *Bordetella* strains (1E7, 1E9, and 2B1) and 2E9, which only binds *B. pertussis* pertactin but binds with much higher affinity than the other member of competition group 4 (Table 1). To more carefully compare binding to bacteria expressing pertactin, we used flow cytometry to calculate effective *K*_*d*_ values under the conditions of excess antibody, as required for Langmuir isotherm analysis (28). Antibodies showed the same affinity ranking as we observed for BLI (Table 1), but with smaller *K*_*d*_ values because of avidity effects of binding the cell surface: from 0.6 to 1.7 nM for TohamaI (Fig. 4*C*) and 1.7 to 5 nM for D420 (Fig. 4*D*). For each antibody, the measured *K*_*d*_ value was about fivefold larger for D420 than for TohamaI, perhaps because of different surface levels of pertactin. The high-level binding to bacterial cells further supports the idea that these antibodies may be relevant for infection.

### Epitopes recognized are immunogenic during infection

**Figure 5:**
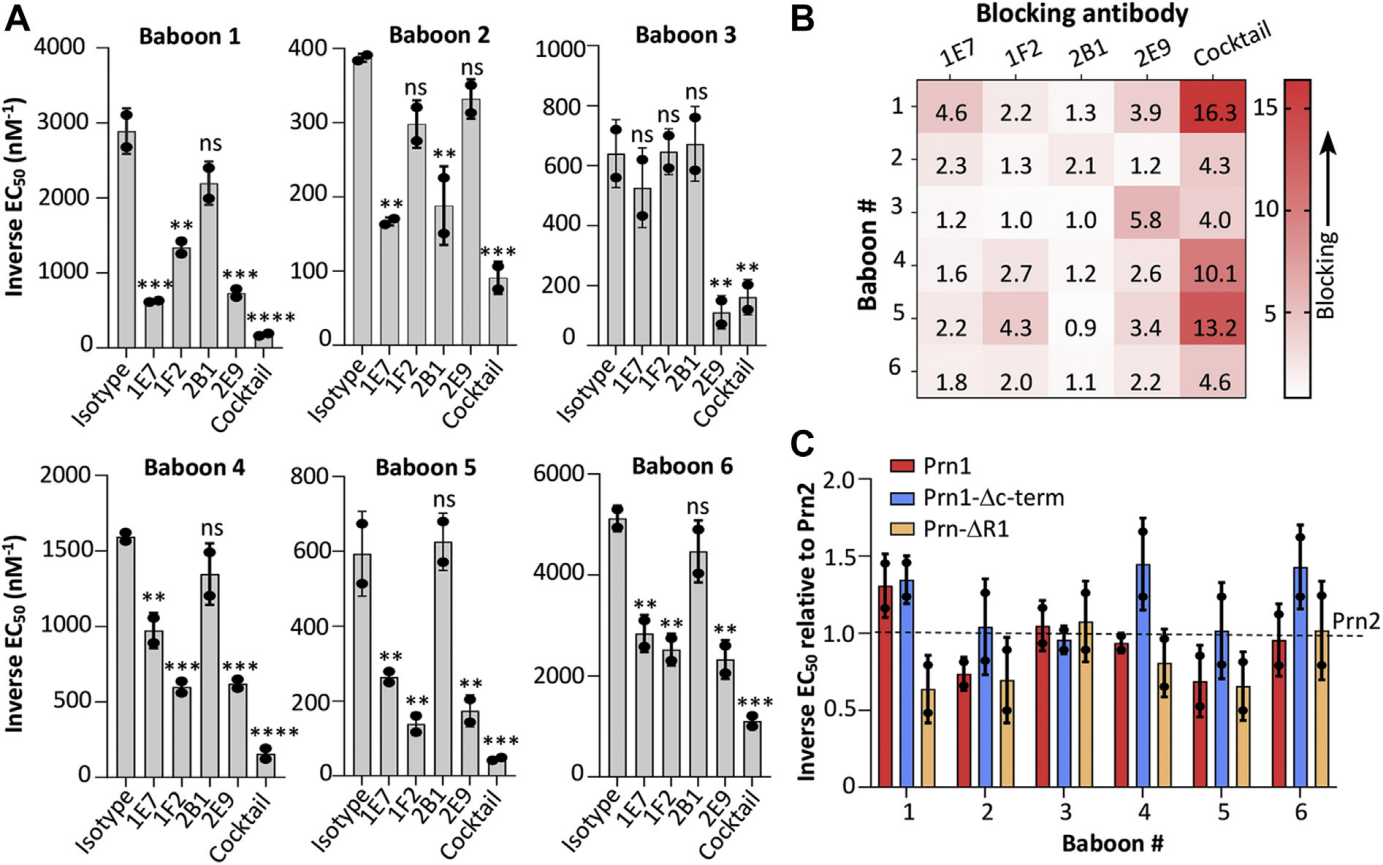
**Antibody epitopes are strongly represented in sera from *Bordetella pertussis*–infected baboons.***A*, ELISA was performed with pertactin-coated plates. After blocking, baboon sera were serially diluted over six steps in duplicate in the presence of a fixed amount of antipertactin antibody or isotype control, each expressed recombinantly with mouse IgG2a/κ constant domains, and detected with antihuman Fc-HRP. GraphPad software was used to determine EC_50_ values; the mean and standard deviation are shown from two independent experiments. *B*, these data are summarized in a heat map showing the fold change in inverse EC_50_ in the presence of each antibody, with *darker red* depicting greater blocking capacity. *C*, binding of baboon sera to Prn1, Prn2, and the constructs Prn-ΔR1- and Prn1-Δc-term-coated ELISA plates, with sera diluted and detected as in *A*. For each Prn variant, the inverse EC_50_ value was determined and normalized to the inverse EC_50_ value for Prn2. Mean and averages of fold changes are from two independent experiments, with no statistically significant differences. Analyses were performed in GraphPad software; ∗*p* < 0.05, ∗∗*p* < 0.01, ∗∗∗*p* < 0.001, and ∗∗∗∗*p* < 0.0001 determined by one-way ANOVA and Tukey's post hoc test to determine *p* values between inverse EC_50_ sera binding blocked by each antibody or cocktail and an isotype control. HRP, horseradish peroxidase; IgG, immunoglobulin G.

To determine whether these antibodies define pertactin epitopes that are immunogenic during infection, we assessed the prevalence of antibodies binding these epitopes in convalescent baboon sera. We analyzed sera from six adolescent baboons, collected 26 days after infection with Prn2-expressing *B. pertussis* D420 when anti-Fha levels were high (29), using a competition ELISA. Prn2-coated plates were incubated with 1E7, 1F2, 2B1, and 2E9 or a cocktail of all four antibodies expressed recombinantly with mouse IgG2a/κ constant domains, before serially diluting sera and detecting captured baboon antibodies. Analysis of EC_50_ values from curves for each blocking antibody, cocktail, or no antibody show that while the cocktail provided 3-fold to 16-fold reduced EC_50_, the impact of individual antibodies varied (Fig. 5*A*). While in most baboons, at least two antibodies significantly reduced sera binding to pertactin, baboon 3 had a polarized response to pertactin, as only 2E9 significantly blocked sera. The antibody 2B1 appeared to be the antibody least represented in the sera, as it only significantly reduced pertactin binding of baboon 2, whereas 2E9 was significant in all baboons except 2 (results summarized in Fig. 5*B*). These results are similar to the 40 and 60% competition with human immune sera to the F fusion protein from respiratory syncytial virus observed for monoclonal antibodies D25 and motavizumab, respectively (30).

To determine the prevalence of antibodies binding epitopes outside the regions recognized by these four antibodies, we assessed sera binding to our four pertactin variants (Prn1, Prn2, Prn-ΔR1, and Prn1-Δc-term) by ELISA. We determined EC_50_ values and normalized these to Prn2, revealing less than twofold differences that were not significant (Fig. 5*C*). This suggests that the epitopes defined by our four antibodies elicited higher titers than epitopes including the R1 and R2 loops. Collectively, these data indicate that these four epitopes identified are immunogenic during infection and can provide insight into the role of antipertactin antibodies elicited during pertussis infection.

### Antibodies protect mice against pertussis infection

**Figure 6:**
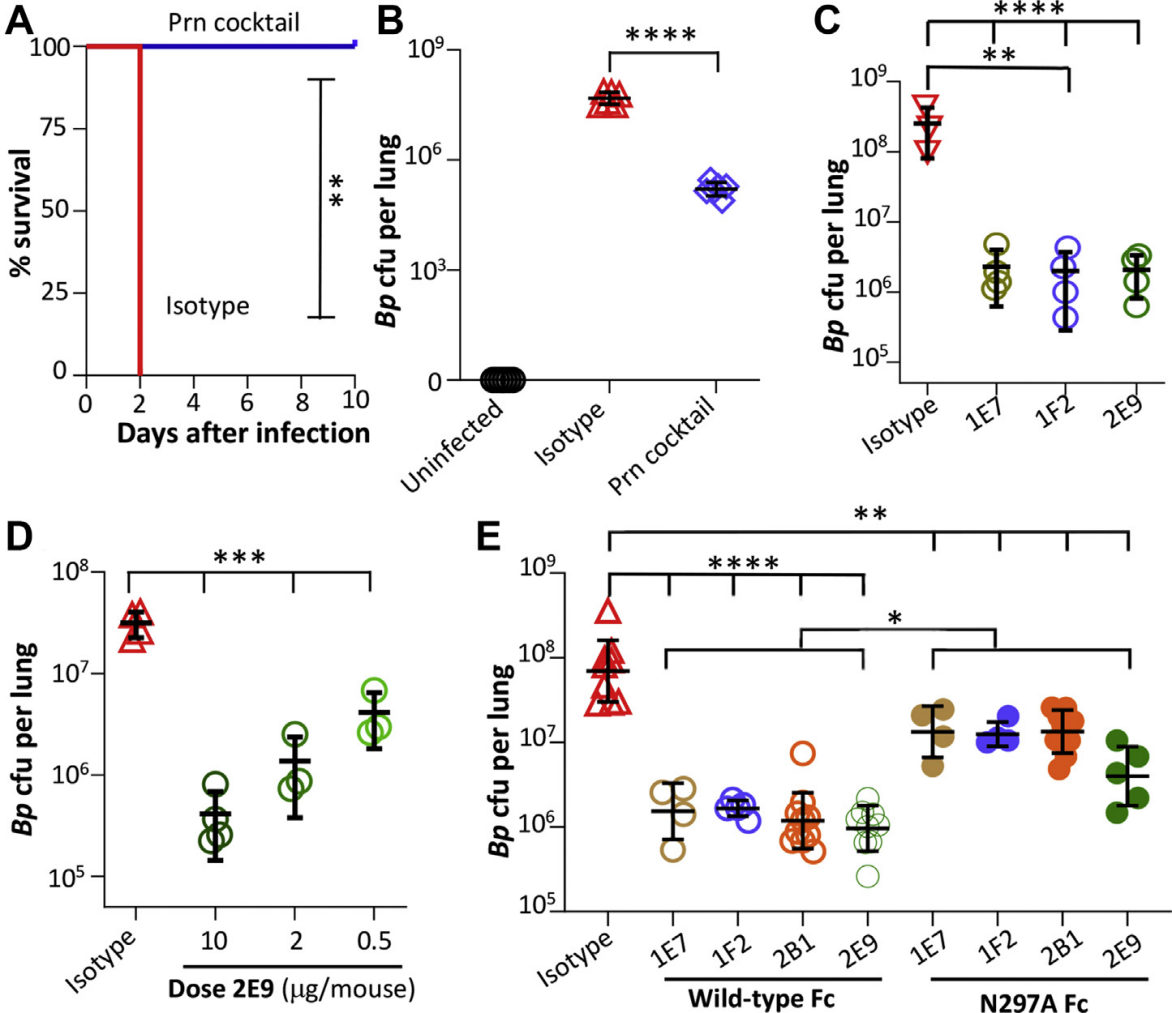
**Antipertactin antibodies protect mice from *Bordetella pertussis* infection.***A*, Balb/c mice (n = 4) were administered an antibody cocktail (7 μg each of 2E9, 2B1, 1E7, and 1F2) or isotype control (10 μg), all as recombinant antibodies expressed with mouse IgG2a/κ constant domains, intranasally before challenge with a lethal dose (2 × 10^8^ cfu) of *B. pertussis* TohamaI and monitored for 10 days. ∗∗*p* < 0.005 determined by Mantel–Cox log-rank test. *B*, mice (n = 6) were administered an antibody cocktail (7 μg each of 2E9, 2B1, 1E7, and 1F2 mouse IgG2a) or isotype control (10 μg) intranasally before challenge with a sublethal dose (2 × 10^8^ cfu) of *B. pertussis* TohamaI, with lung bacterial colonization levels on day 3 postinfection measured by plating of serially diluted lung homogenate. *C*, the experiment in *B*, was repeated with each group receiving the treatment of 1E7, 1F2, 2E9, or isotype (n = 3–5, 10 µg). *D*, the lung colonization experiment was repeated with mice receiving an isotype control (n = 4; 10 μg) or decreasing concentrations of antibody 2E9 (10, 2, and 0.5 μg). *E*, the partially protective dosage of 2 μg was used to compare the lung colonization levels of mice receiving treatments of 1E7, 1F2, 2B1, or 2E9 as wildtype Fc mouse IgG2a (n = 5–10) or mouse IgG2a-N297A (n = 4–9) as well as an mIgG2a isotype control (n = 9). Data pooled from two independent experiments. For all panels, ∗∗*p* < 0.01, ∗∗∗*p* < 0.001, and ∗∗∗∗*p* < 0.0001 determined by one-way ANOVA and Tukey's post hoc test to determine *p* values between groups using GraphPad software. cfu, colony-forming unit; IgG, immunoglobulin G.

Passive antibody protection against *B. pertussis* infection was assessed using a mouse pneumonia model (29, 31, 32). To first determine the impact on survival, mice were intranasally administered a cocktail of antibodies (7 μg each of 2B1, 1F2, 2E9, and 1E7, each expressed recombinantly with mouse IgG2a/κ constant domains) or 10 μg of an isotype control shortly before intranasal challenge with 2.5 × 10^8^ colony-forming unit (cfu) of *B. pertussis* TohamaI. By the second day after infection, all mice receiving the isotype control antibody met euthanasia criteria based on weight loss, similar to prior reports (33). By contrast, all mice receiving the cocktail gained and sustained body weight after day 2 to 3 (Fig. S7) and remained healthy through day 10 and when the experiment was terminated (Fig. 6*A*).

As a more sensitive metric of protection, we next used a lower bacterial load to measure the impact of antibody administration on lung colonization (31). Antibodies (an antipertactin cocktail or isotype control, as aforementioned) were again administered intranasally to mice shortly before infection with 5 × 10^6^ cfu *B pertussis* TohamaI. On day 3 post-infection, mice were sacrificed and lung tissues plated to enumerate *B. pertussis*. No bacteria were recovered from uninfected mice, whereas 5.1 × 10^7^ *B. pertussis* cfu were recovered from mice administered an isotype control, and >100-fold fewer bacteria (1.7 × 10^5^ cfu, *p* < 0.0001) were recovered from mice administered the antibody cocktail (Fig. 6*B*).

To evaluate whether antibodies binding different pertactin epitopes confer different levels of protection, we initially repeated this experiment by administering a single antipertactin antibody or an isotype control at a dose of 10 μg. While 2.5 × 10^8^ cfu were recovered from mice treated with the isotype control, treatment of mice with 2E9, 1F2, or 1E7 reduced colonized ∼100-fold (2–2.2 × 10^6^ cfu; Fig. 6*C*). We then determined the antibody dose–response range by repeating this experiment using antibody 2E9 at 10, 2, or 0.5 μg. While ∼3 × 10^7^ cfu were recovered from mice treated with the isotype control, antibody treatment provided a dose-dependent reduction in colonization, with 10 μg reducing colonization >70-fold and 2 μg providing a significant but not maximal cfu reduction (∼20-fold; *p* < 0.001) and 0.5 μg an even smaller but still significant reduction (approximately eightfold; *p* < 0.001; Fig. 6*D*). To detect differences in antibody protection, we repeated this experiment using a partially protective antibody dose of 2 μg. Whereas mice treated with the isotype had 1 × 10^8^ cfu per lung on day 3, treatment with 1E7, 1F2, 2B1, or 2E9 led to 50-fold to 90-fold reductions in *B. pertussis* lung colonization levels (∼1.8 × 10^6^, 1.7 × 10^6^, 1.7 × 10^6^, and 1.1 × 10^6^ cfu, respectively; *p* < 0.0001; Fig. 6*E*). Antibody thermal unfolding temperatures were similar for all four antibodies and thus unlikely to influence these results (Fig. S6). This suggests that all four antibodies binding multiple epitopes have similar protective capacities.

To better understand the mechanism by which antipertactin antibodies reduce lung colonization by *B. pertussis*, mice were also treated with antibody variants containing a single N297A residue change in the Fc domain in the same experiment with a 2 μg antibody dose. This change eliminates a highly conserved N-linked glycosylation site and greatly impairs Fc effector functions. Specifically, binding of aglycosylated mouse IgG2a Fc to mouse FcγRs is greatly reduced, although binding to C1q is largely retained (34). Mice receiving aglycosylated antibodies exhibited reduced colonization levels compared with the isotype (6–20-fold; *p* < 0.01), which was greatly impaired relative to their wildtype Fc counterparts (*p* < 0.05; Fig. 6*D*). In each case, 2E9 appeared to be slightly, but insignificantly, more protective (90-fold and 20-fold reduced colonization for wildtype and N297A Fc, respectively) despite all four antibodies having similar affinities and epitope accessibilities (Table 1 and Fig. 4*C*). Collectively, these data suggest that protection mediated by antipertactin antibodies is largely Fc mediated, with FcγRs interactions playing a central role in protection.

### Antibodies fail to mediate complement lysis *in vitro*

**Figure 7:**
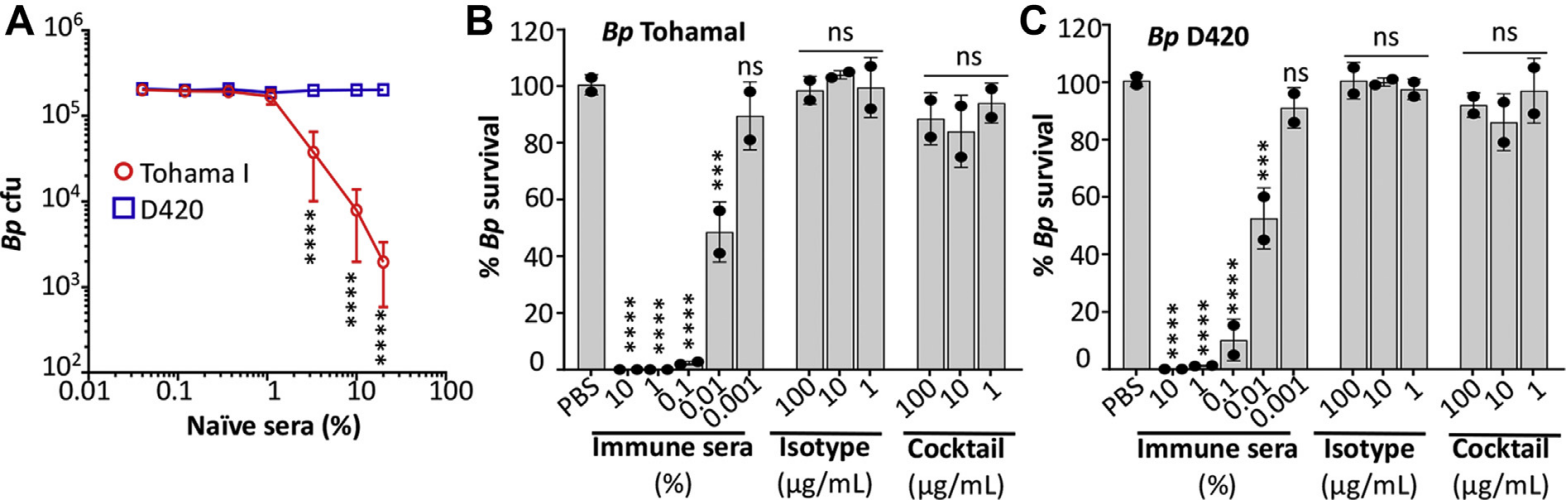
**Antibodies fail to mediate complement lysis *in vitro*.***A*, a complement lysis assay was used to assess susceptibility to naive sera by incubating log-phase bacteria in a 15 μl reaction with HBSS and naive baboon sera as a complement source at various concentrations. After a 2 h incubation at 37 °C, surviving bacteria were enumerated by plating serial dilutions on BG plates. Antibody-mediated complement killing of (*B*) TohamaI and (*C*) D420 was assessed as in *A*, with 10% naive baboon sera as a complement source, and heat-treated convalescent baboon sera or recombinant antibodies were expressed with human IgG1/κ constant domains as an antibody source. The results were normalized with the number of surviving bacteria recovered after incubation with only naive sera set at 100%. Results reported show average of two independent experiments, with two technical replicates each. For each panel, ∗∗∗*p* < 0.001, ∗∗∗∗*p* < 0.0001 determined by one-way ANOVA and Tukey's post hoc test to determine *p* values between groups receiving immune sera, isotype, or cocktail to group receiving PBS. BG, background; HBSS, Hank's balanced salt solution; IgG, immunoglobulin G.

To determine whether complement contributes to the *in vivo* protection observed, we assessed the ability of the antibodies to induce lysis of *B. pertussis* bacteria in an *in vitro* assay. To establish an assay, the susceptibility of *B. pertussis* TohamaI and D420 to naive complement was determined by incubating log-phase cells with serially diluted naive baboon sera (20% to 0.04%) reserved from a previous study (29). After 2 h, surviving bacteria were enumerated by plating on background (BG) plates. While strain D420 resisted killing by up to 20% naive complement, TohamaI was susceptible to complement concentrations higher than 1%. At a concentration of 10% naive sera, surviving TohamaI cells were reduced from ∼2 × 10^5^ to 1 × 10^4^ cfu (*p* < 0.0001, Fig. 7*A*).

To measure antibody-mediated complement killing, a naive serum concentration of 10% was used for all assays that approximate the concentration of complement at human mucosal surfaces and was used in prior studies (26). We compared the ability to promote killing by heat-inactivated convalescent sera from baboons infected with *B. pertussis* TohamaI (29), our four antibodies 1E7, 1F2, 2B1, and 2E9 individually and or combined in an antibody cocktail and an isotype control, all expressed recombinantly with human IgG1/κ constant domains. Convalescent baboon sera promoted efficient killing of TohamaI, as enumerated bacteria decreased from ∼1 × 10^4^ to zero colonies (0% bacterial survival) using immune sera concentrations of 10% and 1%. Colonies were observed when diluting immune sera to 0.1% or lower, with ∼50% bacteria killing when immune sera were diluted to 0.01%. In contrast, using our isolated antibodies binding different epitopes individually or combined, no bacterial killing was observed, similar to the isotype control, suggesting that these antibodies are unable to mediate complement-dependent cell killing (Figs. 7*B* and S8). These data, along with the mouse colonization showing impaired protection by aglycosylated antibodies, support an important role for FcγR interactions in mediating protection by antipertactin antibodies.

## Discussion

Despite the presence of pertactin in acellular vaccines for >20 years and the apparent ability of antipertactin antibodies to drive pertactin variation and loss, little is known about pertactin antigenicity or its role in pathogenesis (35). This is partly because of the few monoclonal antibodies binding pertactin that have been described, which has limited investigations into pertactin structure–function correlates. Accordingly, we sought to identify antibodies binding diverse pertactin epitopes and use these to begin understanding mechanisms of antibody protection against pertussis. We characterized a panel of 12 antibodies binding four distinct pertactin epitopes, with four antibodies binding epitopes conserved across all three classical *Bordetella* strains.

Pertussis produces a number of large and beta-helical proteins with repeat regions that are implicated in virulence, including Fha (36), the adenylate cyclase toxin (ACT) (37) and pertactin (8). There is little structural information for these proteins alone or in complex with antibodies, but structures of two neutralizing antibodies complexed with an ACT fragment were recently reported. These showed that both antibodies bind linkers between beta-roll repeats and suggested that exposed loops in other pertussis antigens may also mediate antibody- and protein-binding interactions (37). In accordance with this hypothesis, pepscan analysis of mouse monoclonal antibodies elicited by Prn1 immunization determined that the linear R1 and R2 loops were commonly recognized (38). Further epitope mapping with pertactin truncations, deletions, and residue substitutions showed that the R1 and R2 loops can be components of larger conformational epitopes, and that additional epitopes exist outside of R1 and R2 (19). Apart from epitope analyses, these studies report limited biochemical data, such as affinity, epitope conservation, or functional activity. Interestingly, of the antibodies we characterized, none recognize the R1 or R2 regions as binding was not impacted by removal of these regions from pertactin (Fig. 2*A*). In addition, antibodies binding these four epitopes are present in convalescent baboon sera (Fig. 5*A*), whereas antibodies binding epitopes involving R1 or R2 appeared less common in these sera (Fig. 5*C*). These data suggest the presence of conserved immunogenic epitopes in pertactin that are independent of R1 and R2, a different result than that observed for ACT, and many previously reported antipertactin monoclonal antibodies.

The roles of the R1 and R2 variable repeats and the forces driving their diversification, which differentiates pertactin alleles and homologs, remain open questions. The shift from Prn1 to Prn2 in clinical isolates, with different numbers of repeats in R1 (Fig. 1*B*), occurred after the widespread introduction of whole-cell vaccines containing small amounts of pertactin. If the R1 and R2 repeats are highly immunogenic, then selective pressures exerted by low antibody levels could drive diversification in this region to escape antibody binding. Antigenic phase variation is seen in several other bacterial surface proteins but usually occurs within the context of a single infection such as for the *S. pneumoniae* PspC protein (39), whereas the timescale of R1 and R2 variation in pertactin is much slower. The R2 region is particularly interesting, as it is unresolved in the crystal structure, which is not surprising considering proline-rich repeats are often “loosely” structured (40). The *Streptococcus mutans* P1 adhesin protein, which includes three proline-rich repeats that form an extended stalk structure with a polyproline type II helix (41), suggests a possible similar role in pertactin. However, none of the antibodies identified here exhibit selective binding to Prn1 or Prn2 or engage R1 or R2 sequences (Fig. 2*A*). It may be that antibodies binding R1 and R2 were not identified in this study because of biases in our affinity-driven selection process, or they may be better induced by whole-cell vaccination than acellular vaccination or infection (Fig. 5). Future efforts will aim to identify antibodies binding the R1 and R2 regions to better define their roles.

By virtue of its close association with the bacterial membrane, pertactin is thought to be a good target for antibody-mediated complement lysis and phagocytosis of *B. pertussis* (42). Sera from individuals immunized with the acellular vaccine have shown strong correlations between pertactin titers and opsonophagocytic activities in *in vitro* assays (7, 43). Additional experiments with polyclonal IgG purified from human immune sera promotes phagocytosis through the activating Fc receptor FcγRIIa on human polymorphonuclear cells *in vitro* (44). The ability of pertactin-containing vaccines to promote complement killing is less apparent: some serological studies show an effect (4, 45), whereas others do not (46, 47). This could be due to procedural differences, as different complement sensitivities are exhibited by different *Bordetella* strains (Fig. 7*A*) (48), growth phases (49), and complement sources (45). However, monoclonal antibodies and immune sera binding the *B. pertussis* lipooligosaccharide reliably mediate complement lysis (50).

Surprisingly, antibodies binding at least five pertactin epitopes are capable of mediating bacterial clearance. This contrasts with the *S. pyogenes* M protein in which only the antigenically variable aminoterminus elicits antibodies that are both opsonic and protective in murine models and correlate with protection in humans. It also suggests that the variable repeats serve different functions in pertactin than in the M protein, where they evade binding by serum proteins (20). One previous antipertactin mAb, PeM4, which binds a linear R1 epitope, was evaluated for passive protection of mice. Treatment with a large antibody dose (250 μg) reduced *B. pertussis* lung colonization by ∼10-fold on day 3 postinfection (18). We showed that treatment with a cocktail of four antibodies—all expressed as recombinant mouse IgG2a/κ antibodies—protected mice against lethal infection and, in separate studies, reduced *B. pertussis* lung colonization by ∼100-fold on day 3 after infection when administered at a modest 10 μg dose (Fig. 6, *A*–*C*). Administration of a partially protective 2 μg antibody dose impaired protection by antibodies bearing aglycosylated Fc domains with reduced binding to mouse FcγRs (51) (Fig. 6, *D* and *E*). Coupled with the lack of *in vitro* complement killing by any of these antibodies (Fig. 7*C*), opsonophagocytosis is strongly implicated as mediating *in vivo* protection.

Pertactin has been proposed to possess several functions contributing to pathogenesis, including bacterial adhesion and immunomodulation, while the repeat regions have been speculated to serve immune-evasive functions. Accordingly, we expected to find antibodies with different protective capabilities because of disruption of different pertactin functions. Instead, it appears that many antibodies binding surface-exposed pertactin epitopes can mediate bacterial clearance (Fig. 6). This may explain why whole-cell vaccines containing small amounts of pertactin and eliciting low levels of antipertactin antibodies could drive antigenic shift from Prn1 to Prn2, whereas acellular vaccines containing large amounts of pertactin (3–8 μg) elicit high titers of polyclonal antipertactin antibodies, many of which are bactericidal, and provide strong selective pressures for antigen loss (52). The bactericidal effects are so strong in our mouse colonization model that they obscure other possible antibody-mediated effects. However, protection by low doses of N297A antibodies with impaired Fc effector functions provides hints that these antibodies may mediate effects in addition to cellular killing. Specifically, the four antibodies used here have very similar affinities (0.6–1.73 nM for TohamaI bacteria; Fig. 4*C*), identical constant domains (mouse IgG2a/κ), and similar stability profiles (melting temperatures 62–66 °C; Fig. S6). Accordingly, any differences in protection are expected to be due to the epitopes recognized. Our data suggest that 2E9 confers superior protection as an aglycosylated antibody (Fig. 6*E*), which could be due to blockade of pertactin functions that support pathogenesis. The possibility that 2E9, or other antibodies, block pertactin activities will be pursued in future work.

In summary, we developed a panel of antipertactin antibodies binding distinct epitopes that individually mediate *B. pertussis* clearance after experimental infection of mice. Pertactin's contributions to infection are unclear but have been proposed to include bacterial adhesion to eukaryotic cells *via* RGD motifs (15, 16) and immunomodulatory activities to resist neutrophil clearance (3, 17, 52). A role in adhesion has been particularly difficult to determine because of the presence of multiple adhesins (Fha, Fim2/3, and perhaps others) with overlapping functions (9, 53, 54). Accordingly, future work will determine the epitopes recognized by these antibodies with molecular resolution and use them to further define pertactin's role(s) in supporting *Bordetella* infection and the impact of pertactin immunization on bacterial fitness.

## Experimental procedures

### *Bordetella* strains and bacterial growth

*B. pertussis* strains TohamaI, D420, H973 (catalog no.: NR-42460; BEI Resources), I176 (catalog no.: NR-42463; BEI Resources), H921 (catalog no.: NR-42457; BEI Resources), *B. bronchiseptica* RB50, and *B. parapertussis* 12822 were used in this study. Cells were grown on Bordet–Gengou agar plates supplemented with glycerol and 15% defibrinated sheep's blood (hemostat) for 3 days at 37 °C. Liquid cultures were grown in fully supplemented Stainer–Scholte media (SSM) (41). Challenge inoculum was prepared by inoculating plate grown bacteria into liquid culture for overnight growth at 37 °C and 225 rpm. The following day, cells were concentrated approximately fivefold using a tabletop centrifuge (1500*g* for 30 min). Cells were resuspended in the remaining SSM, and 100% sterile glycerol was added to a final 15% v/v before freezing 200 μl aliquots at −80 °C. The following day, one aliquot was thawed, serially diluted in PBS, and plated dropwise on BG plates in triplicate to determine the initial cfu/ml concentration.

### Pertactin production

Expression of pertactin from inclusion bodies was performed as previously described (55, 56). The mature Prn1 and Prn2 sequences (residues 35–637 and 35–642, respectively, lacking the signal sequence and porin domain) and variants Prn1-ΔR1 with R1 replaced by a (Gly_4_Ser)_2_ glycine–serine linker and Prn1-Δc-term, with the C-terminal 67 residues truncated were PCR amplified from TohamaI genomic DNA with an N-terminal Strep tag and cloned into plasmid pET28a+ *via* NheI and BamHI restriction sites. *E. coli* BL21(DE3) starter cultures containing the plasmid were grown overnight in the presence of 1% glucose and then diluted 1:100 into 500 ml cultures with 1% glucose and kanamycin and grown overnight. In the following day, the cultures were induced with 1 mM IPTG at 37 °C for 5 h. Cells were harvested by centrifugation and resuspended in PBS with a protease inhibitor cocktail (Sigma) before sonication (Qsonica; 80%, 1:10 dilution, 10 s on, 20 s off) to lyse the cells. After centrifugation for 30 min at 22,000*g*, the pellet was washed twice (50 mM Tris, 5 mM EDTA, 2 M urea, 2% Triton X-100, and pH 8.0), followed by centrifugation at 22,000*g* for 20 min. Three more washes were performed with 50 mM Tris (pH 8.0) to remove detergent.

The pellet was then resuspended with 8 M urea, 50 mM Tris, 100 mM NaCl, 0.2 mM CaCl_2_, pH 8.0, and centrifuged at 20,000*g* for 30 min to clarify the extract. The urea concentration was adjusted to 6 M and applied to a Strep-Tactin (IBA) column on an Akta FPLC. After washing, bound protein was eluted with 50 mM biotin + Hepes-buffered saline with 2 mM calcium in 6 M urea, pH 8.0. Fractions containing eluted protein were pooled and dialyzed (20-fold volume excess) against 50 mM Tris, 100 mM NaCl, pH 8.0, and 0.4 M l-arginine overnight at 4 °C and then the same buffer without l-arginine for two more days. Recovered protein was concentrated with Amicon centrifugal filter units before a size-exclusion chromatographic polishing step using Superdex S200 (Akta). SDS-PAGE (4–20% gradient gel; Bio-Rad) was used to assess pertactin purity and molecular weight.

### Phage scFv library generation and panning

ScFv phage display libraries were generated as described (23). Briefly, mRNA was extracted from homogenized spleens using PureLink RNA kit (Invitrogen), and complementary DNA was generated using Superscript IV (Invitrogen). Variable heavy and light chain domains were amplified using a cocktail of primers described by Krebber *et al.* (23) and joined into scFvs with optimized overlapping primers that encode a (Gly_4_Ser)_4_ linker with four repeats. The assembled genes were amplified, digested with SfiI, gel purified, and ligated en masse into similarly digested phagemid pMopac24 (100 μg). Ligation reactions were processed using DNA Clean & Concentrator Kit (Zymogen) and electroporated into XL1Blue *E. coli* cells in 12 transformations, each recovered with 1 ml Super optimal broth with Catabolite suppression media before plating on 2xYT with 1% glucose and 200 μg/ml ampicillin. Dilutions of the transformations were used to estimate library size, with vector-only ligations used to assess BG. Cells were scraped from plates with media, adjusted to 25% glycerol, and stored at −80 °C.

For phage production, library cells were added to 30 ml 2xYT for a starting absorbance of 0.08 at 600 nm in the presence of 200 μg/ml ampicillin and 1% glucose and gown with shaking at 37 °C until reaching an absorbance of 0.5 at 600 nm. Cells were infected with 1E10 pfu/ml M13KO7 helper phage and expression of the scFv-gpIII fusion induced by 1 mM IPTG. After 2 h at 37 °C, 50 μg/ml kanamycin was added, and phage production allowed to continue overnight with shaking at 25 °C. To amplify phage after panning rounds, recovered phage (200 μl) was used to infect 2 ml exponential phase XL1Blue cells at an absorbance of 0.5 at 600 nm for 30 min at 37 °C. The culture was added to 30 ml 2xYT media with 1% glucose and 200 μg/ml ampicillin, and phage production continued, as aforementioned.

Phage was purified from overnight cell cultures by double precipitation. The cell culture supernatant was harvested by centrifugation at 8000 rpm for 15 min. The supernatant was then combined with one-fifth volume precipitation buffer (2.5 M NaCl and 20% PEG-8000) and incubated at room temperature for 5 min and then on ice for ∼1 h. Phage was pelleted by centrifugation at 8000 rpm for 10 min, thoroughly decanted, and the pellet resuspended in 1 ml PBS. Phage was enumerated by titration and stored at in 50% glycerol at −20 °C.

Phage panning was performed on pertactin-coated and blocked 96-well plates. Pertactin coating concentration was reduced each round, from 5, 4, 3, and finally 2 μg/ml and blocked with PBS containing 2% bovine serum albumin (BSA) or 10% milk. Phage was diluted with an equal volume of PBS with 0.05% Tween-20 (PBS-T) and 2% BSA or 10% milk, and 160 μl added to eight wells for 1 h and then washed extensively by vigorously pipetting (>200 times) with PBS-T. Bound phage was eluted with 50 μl/well, 0.1 N HCl, pH 2.2 (pH adjusted with glycine) for 10 min at room temperature, transferred to new tubes, and neutralized with 6% volume 2 M Tris buffer (3 μl neutralization buffer per 50 μl elution from each well). Recovered phage was enumerated by titration. Antibody sequences were determined by sequencing phagemids recovered from individual colonies.

### Serological, phage, and antibody ELISA

For serological analyses, recombinant protein characterization, and phage ELISAs, pertactin or anti-c-myc (9E10) were coated in PBS, at 3 or 2 μg/ml, respectively, in 96-well adhesive ELISA plates (Corning) overnight at 4 °C. Uncoated plates were included as controls. Plates were blocked with 5% milk or 2% BSA in PBS-T (0.1% Tween-20) for 1 h prior to the addition of serum or antibodies first diluted in milk or equivolume diluted phage in duplicate. Samples were then serially diluted fivefold down the plate and incubated for 1 h at room temperature with rocking. Plates were washed three times with PBS-T between each subsequent step. To detect bound antibodies or phage, antimouse Ig-horseradish peroxidase (HRP) (Southern Biotech) or anti-M13-HRP (Santa Cruz) secondary antibody was added at a 1:2000 dilution in blocking buffer and incubated at room temperature for 1 h. Signal was detected with 50 μl TMB substrate (Pierce) and quenched with equal volume 1 N HCl. Absorbance values were read at 450 nm on a SpectraMax M5 plate reader and analyzed in GraphPad Prism 7 (GraphPad Software, Inc).

### Antibody production

ExpiCHO-S cells were transfected using the high-titer protocol according to the manufacturer's instructions. Briefly, cells grown to 7 to 10 × 10^6^/ml with >95% viability were diluted to 25 ml at 6 × 10^6^/ml. Purified DNA plasmids were diluted in OptiPRO and incubated with ExpiFectamine diluted in OptiPRO for 5 min to allow formation of complexes. The DNA–ExpiFectamine mixture was added to cells overnight at 37 °C with 8% CO_2_ with orbital shaking at 125 rpm. About 20 h later, ExpiCHO feed plus ExpiCHO enhancer was added, and cells were moved to 32 °C, 8% CO_2_ with shaking for 10 to 12 days. Antibodies were purified from supernatant using a 1 ml HiTrap Protein A column (GE) on an ÅKTA-FPLC. The column was washed with 10 column volumes of binding buffer (25 mM Tris, 25 mM NaCl, and pH 7.4) and eluted with 10 column volumes acidic elution buffer (100 mM sodium citrate, 150 mM NaCl, and pH 2.3). Eluted fractions were pooled and buffer exchanged into PBS with 10 kDa molecular weight cutoff Amicon centrifugal filter units and stored at 4 °C.

### Apparent *K*_*d*_ measurements and epitope binning by BLI

Equilibrium *K*_*d*_s of IgGs were measured using the Octet Red96 (ForteBio) instrument. Antihuman IgG Fc (AHC) (ForteBio) sensors were loaded with antibodies in kinetic buffer (0.01% BSA and PBS-T [0.002% Tween-20]) at 10 nM until a response of 0.4 nm was reached. Association curves were recorded for 1 min by incubating the sensors in different concentrations of pertactin, starting from 125 nM, and serial 1:2 dilutions. Dissociation step was recorded for 5 min in the kinetic buffer. Steady-state *K*_*d*_ values were determined using the response values obtained after 5 min of association using the Octet analysis software. To determine if antibodies can simultaneously bind pertactin, mouse antibodies were loaded into antimouse IgG Fc (AHC) sensors (ForteBio) to a response of 0.4 nm followed by a baseline step. Next, the antibody was dipped into pertactin at 60 nM, followed by a baseline step. Finally, sensors with antibody–pertactin complex were dipped into wells, each containing human versions of antibodies being tested. Response derived from the second antibody binding was recorded. Controls included dipping sensors coated with human antibodies into wells with human antibodies without pertactin.

### Surface plasmon resonance

The purified chimeric 2E9 was immobilized on a CM5 sensor chip (GE Healthcare) *via* 1-ethyl-3-(3-dimethylaminopropyl)carbodiimide/*N*-hydroxysuccinimide coupling using a sodium acetate buffer at pH 4.0 for a total of 600 response units. A blank flow cell was used as the reference channel. Pertactin was injected starting at 250 nM followed by twofold dilutions at 30 μl/min for 3 min and allowed to dissociate for 4 min at 25 °C. Regeneration was performed using 10 mM glycine buffer at pH 1.5. On-rate, off-rate, and equilibrium-binding analyses were performed using BIAEvaluation 3.0 software (Biacore) and fit using the 1:1 Langmuir binding model. All injections were performed twice, and final kinetic values reported are the average and standard deviation for the entire dataset.

### Antibody binding to *Bordetella* cells

To assess binding to the various *Bordetella* strains, cells were streaked from −80 °C stocks into blood agar plate and grown at 37 °C for 3 days. Several streaked colonies were grown in liquid media (Strainer and Scholte, fully supplemented) at 37 °C overnight. Following day, cells were washed, and absorbance at 600 nm was adjusted to 1. Approximately 1 × 10^6^ cells were used for staining in a staining volume of 50 μl. Staining was performed with purified antibodies at 10 μg/ml in PBS + 1% BSA on ice for 60 min. Cells were then washed two times in PBS + 1% BSA (centrifugation at 6000*g* for 4 min). Secondary antibody goat–antihuman Fc-Alexa Fluor 647 (Jackson Laboratories) was incubated on ice for 40 min in 50 μl. Cells were washed again and resuspended in 500 μl PBS, placed on ice, and samples were analyzed using Fortessa Flow Cytometer. To determine effective *K*_*d*_ to TohamaI and D420, serial dilutions of antibodies starting from 27 μg/ml following serial 1:3 dilutions were incubated with bacteria at room temperature for 60 min in PBS + 1% BSA and then stained as described previously. To calculate effective *K*_*d*_ values, the geometric mean of the goat–antihuman Fc-Alexa Fluor 647 was fit using GraphPad Prism 7.03. One-site specific binding equation was used (Y = Bmax ∗ X/(*K*_*d*_ + X)) as described (28). The *K*_*d*_, standard error, and *R*^2^ are reported.

### Western blots

To assess binding of the antibodies by Western blot, 100 ng of purified pertactin-1 were boiled and ran by SDS-PAGE gel. The gel was transferred to polyvinylidene fluoride membranes and blocked with PBS-T with 5% milk for 1 h at room temperature. Isolated antibodies were incubated with membrane at 0.5 mg/ml in the blocking solution for 1 h. After several washes, membranes were incubated with antihuman IgG Fc-HRP (SouthernBiotech) at 1:8000 dilution, for 1 h at room temperature. After several washes, membranes were developed using SuperSignal West Pico Chemiluminescent Substrate (Thermo Scientific) and imaged. Western blot with 3G4 was performed by running 100 ng of purified Prn1 and Prn2, and *B. pertussis* TohamaI and D420, *B. bronchiseptica* and *B. parapertussis* cells that were grown overnight, washed, and 10 μl of cells were used after an absorbance at 600 nm was adjusted to 4.

### Protein thermal unfolding

Protein Thermal Shift Dye Kit was used to measure thermal unfolding of antibodies as mouse IgG2a. Fluorescence measurements (λ_ex_ = 580 nm, λ_em_ = 623 nm) were performed using a ThermoFisher ViiA 7 Real-Time PCR System, with a temperature ramp rate of 0.05 °C/s, starting from 25 to 99 °C.

### Baboon sera analysis

To measure the blocking capacity of each antibody representative of each competition group, Prn2-coated plates were first incubated with isotype, 1E7, 1F2, 2B1, 2E9, or a cocktail of all four antibodies (2 μg/ml each) as mouse IgG2a for 1 h at room temperature. Then sera from six different baboons (29) 26 days after infection with *B. pertussis* D420 were serially diluted (starting 1:3 and doing additional six 1:5 dilutions) and incubated for 1 h at room temperature. Sera antibodies were detected with antihuman IgG-Fc HRP (Southern Biotech). To measure baboon sera binding to Prn1, Prn2, Prn1-Δc-term, and Prn1-ΔR1, plates were coated with each pertactin variant at 2 μg/ml overnight at 4 °C, and ELISAs were performed as aforementioned. The inverse EC_50_ to each pertactin variant was determined, and the EC_50_ fold change to Prn2 was reported. Experiments were repeated once.

### Mouse immunization and infection

All animal procedures were performed in a facility accredited by the Association for Assessment and Accreditation of Laboratory Animal Care International in accordance with protocols approved by UT Austin (2018-00092 and 2017-00258) Animal Care and Use Committees and the principles outlined in the *Guide for the Care and Use of Laboratory Animals*.

For immunization, Balb/c mice (6 weeks old, n = 3) received a subcutaneous primary immunization with 8 μg of commercial pertactin (catalog no.: NR-34571; BEI) diluted in PBS with equal volume of complete Freund's adjuvant (Sigma). Four weeks later, mice were similarly boosted with incomplete Freund's adjuvant (Sigma). Two weeks after boosting, pertactin-specific titers were determined by ELISA using tail vein blood samples. Mice were then sacrificed by cardiac puncture and cervical dislocation, and spleens were harvested and placed in RNAlater.

For lethal infection, 32-day-old female and male Balb/c mice were sedated with 60 mg/kg ketamine and 8 mg/kg xylazine anesthesia. Once nonresponsive to toe pinch, mice were administered a cocktail of antipertactin mouse IgG2a mAbs (7 μg each 2E9, 1F2, 1E7, and 2B1) or isotype control in 5 μl per nare and then challenged with 25 μl ∼2.5 × 10^8^ cfu TohamaI per nare. Mice were placed on a heating pad and monitored until regaining consciousness and returned to their cages. Welfare checks were made approximately five times per day to monitor health status and weight. At the conclusion of the experiment, mice were sacrificed by CO_2_ asphyxiation and cervical dislocation.

For sublethal infection, experiments were performed as aforementioned but with ∼5 × 10^6^ cfu *B. pertussis* TohamaI per mouse. Three days after infection, mice were euthanized with CO_2_, and cervical dislocation and the lungs dissected into PBS and homogenized with dounce tissue grinders. To enumerate the bacteria, homogenized tissue was serially diluted in PBS, and 7 μl drops were plated on Bordet–Gengou agar plates and grown at 37 °C for 3 days.

### Complement-dependent cytotoxicity assay

Naive sera as a complement source and convalescent baboon sera were obtained from a previous study (29). To inactivate complement, samples were heated at 56 °C for 30 min. Bacteria were prepared by streaking cells on blood agar plated and incubating 3 days at 37 °C. Next, liquid cultures were started from streaked cells, in 2.5 ml of complete SSM medium (with heptakis) with starting absorbance of 0.1 at 600 nm and grown into midlog phase at 37 °C for 5 h (absorbance at 600 nm = ∼0.25).

To assess susceptibility to complement only, 1.5 μl of cells (after adjusting absorbance of to 0.18 at 600 nm in Hank's balanced salt solution [HBSS]) were incubated with different concentrations of active or inactive naive sera diluted in HBSS (final volume of 15 μl) and incubated at 37 °C for 2 h in a thermocycler. The reaction was quenched by adding EDTA to 10 mM. Samples were serially diluted, and 7 μl drops of dilutions were plated in triplicate on BG plates and grown for 3 days at 37 °C. Cfus were counted and multiplied by the dilution factor to enumerate surviving bacteria.

To evaluate antibody-mediated complement killing, 1.5 μl of bacteria (absorbance of 0.018 at 600 nm) was initially mixed with either immune sera, purified pertactin antibodies, or PBS for 15 min at room temperature in a final volume of 13.5 μl with HBSS. Next, 1.5 μl of naive baboon serum (complement source) was added to all reactions (10%, final volume of reaction = 15 μl) and incubated at 37 °C for 2 h in a thermocycler. The reaction was quenched by adding EDTA to 10 mM. Samples were serially diluted, and 7 μl drops of dilutions were plated in triplicate on BG plates and grown for 3 days at 37 °C. Cfus were counted and multiplied by the dilution factor to enumerate surviving bacteria. To calculate percent survival, 100% survival was given to cells incubated with active complement and PBS. All reactions were performed twice, with freshly streaked bacteria.

## Data availability

Raw data will be made available upon reasonable request.

## Supporting information

This article contains supporting information.

## Conflict of interest

The authors declare that they have no conflicts of interest with the contents of this article.
