# Supplementary material for: Antibodies binding diverse pertactin epitopes protect mice from Bordetella pertussis infection
Source: J Biol Chem. 2022 Feb 11;298(3):101715. doi: 10.1016/j.jbc.2022.101715 (PMC8931430; doi:10.1016/j.jbc.2022.101715)
Supplement: Supplemental Figures S1–S8 [file mmc1.docx]

**Antibodies binding diverse pertactin epitopes protect mice from *B. pertussis* infection**

Rui Silva^1^, Andrea M. DiVenere^2^, Dzifa Amengor^1^, Jennifer A. Maynard^1,2,#^

^1^Department of Molecular Biosciences, University of Texas, Austin, Texas, USA

^2^Department of Chemical Engineering, University of Texas, Austin, Texas, USA

#Address correspondence to Jennifer Maynard, [maynard@che.utexas.edu](mailto:maynard@che.utexas.edu)

**Supplemental Information:**

**Figure S1** Discovery of diverse anti-pertactin antibodies.

**Figure S2.** Purification of antibody variants as human IgG/kappa IgG.

**Figure S3.** Antibodies bind pertactin with low nanomolar affinities.

**Figure S4.** Antibodies bind cell-associated pertactin.

**Figure S5.** Only antibody 3G4 detects pertactin on a Western blot.

**Figure S6.** Antibody thermal unfolding.

**Figure S7.** Weight changes from lethal mouse challenge in Fig.6B

**Figure S8.** Antibody-meditated complement dependent lysis of *Bp*

**Figure S1. Discovery of diverse anti-pertactin antibodies. A,** Phylogenic tree of isolated antibodies constructed using the heavy and light variable regions with the Genius software. **B,** Germline heavy and light chain variable region gene usage, number of somatic hypermutation changes observed and CDRH3 and L3 sequences for each characterized antibody.


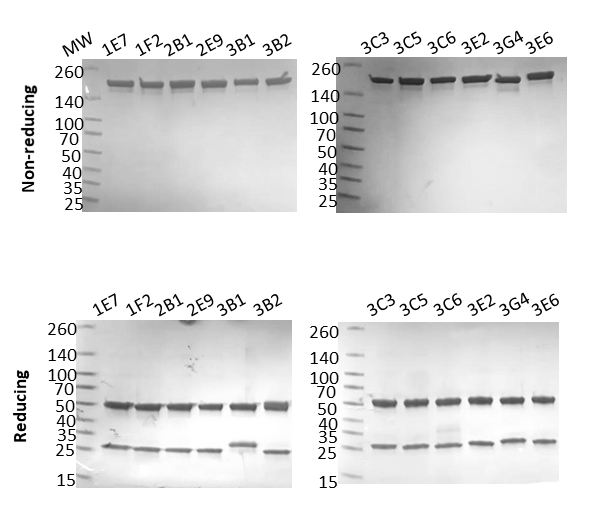


**Figure S2. Purification of antibody variants as human IgG/kappa IgG.** Analysis of protein A-purified protein by 4-20% gradient SDS-PAGE under reducing and non-reducing gel was used to assess protein purity and molecular weight.


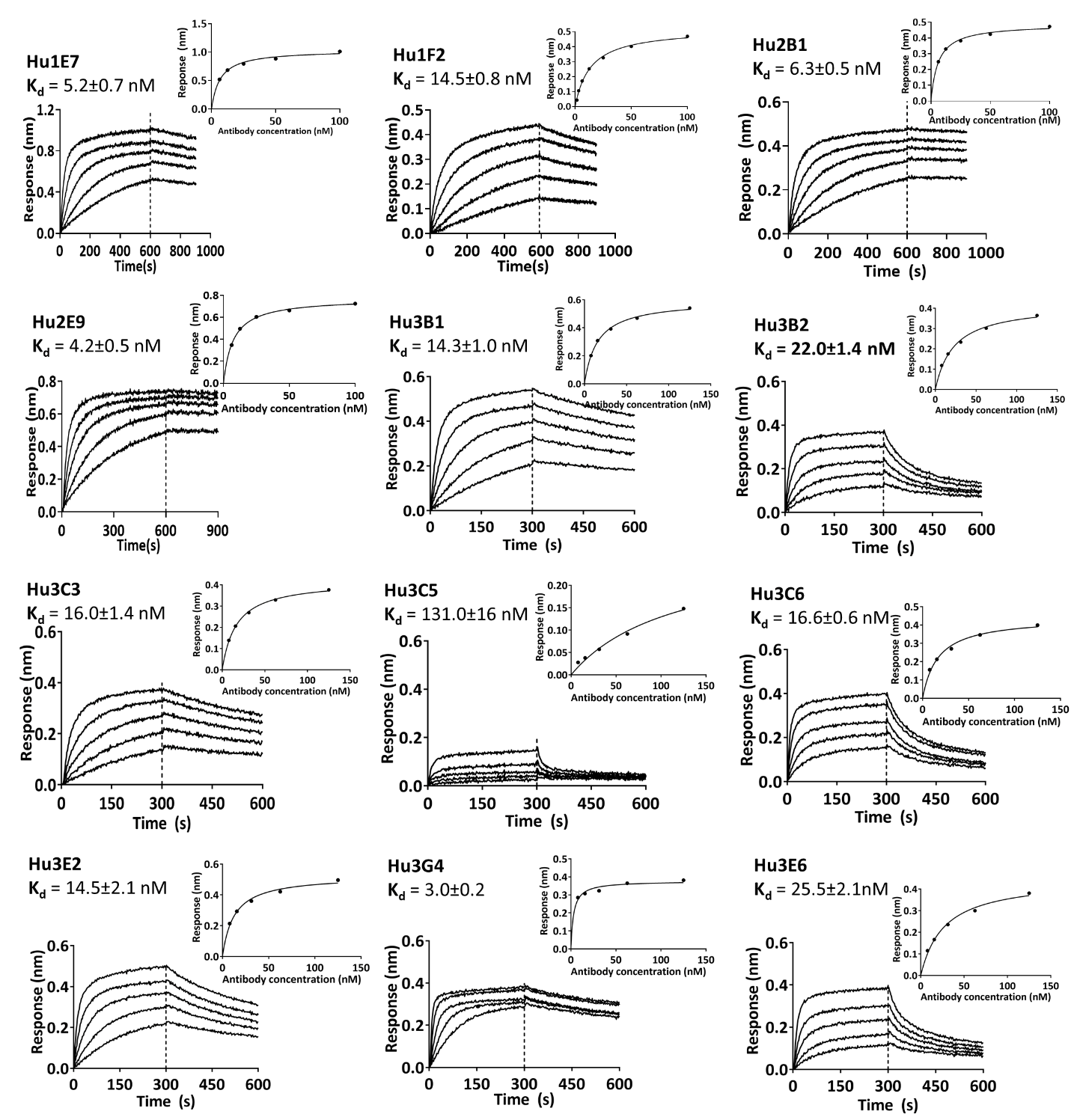
**Figure S3. Antibodies bind pertactin with low nanomolar affinities.** Anti-human Fc BLI sensors were coated with purified antibodies to a 0.4 nm response, then dipped into wells containing pertactin (five 2-fold serial dilutions from 125nM to 0.98 nM) for five minutes to allow for association and then dipped into wells with buffer only to allow for five minutes of dissociation (for antibodies 1E7, 1F2, 2B1 and 2E9, 1:2 serial dilutions started at 100nM and association was recorded for 10 minutes). Equilibrium binding K_d_ values were determined using the response values after five minutes of association using a 1:1 Langmuir binding model with Octet analysis software. Experiment was repeated one time. Values reported correspond to mean and standard deviation of two experiments. Representative curves from one experiment are shown.


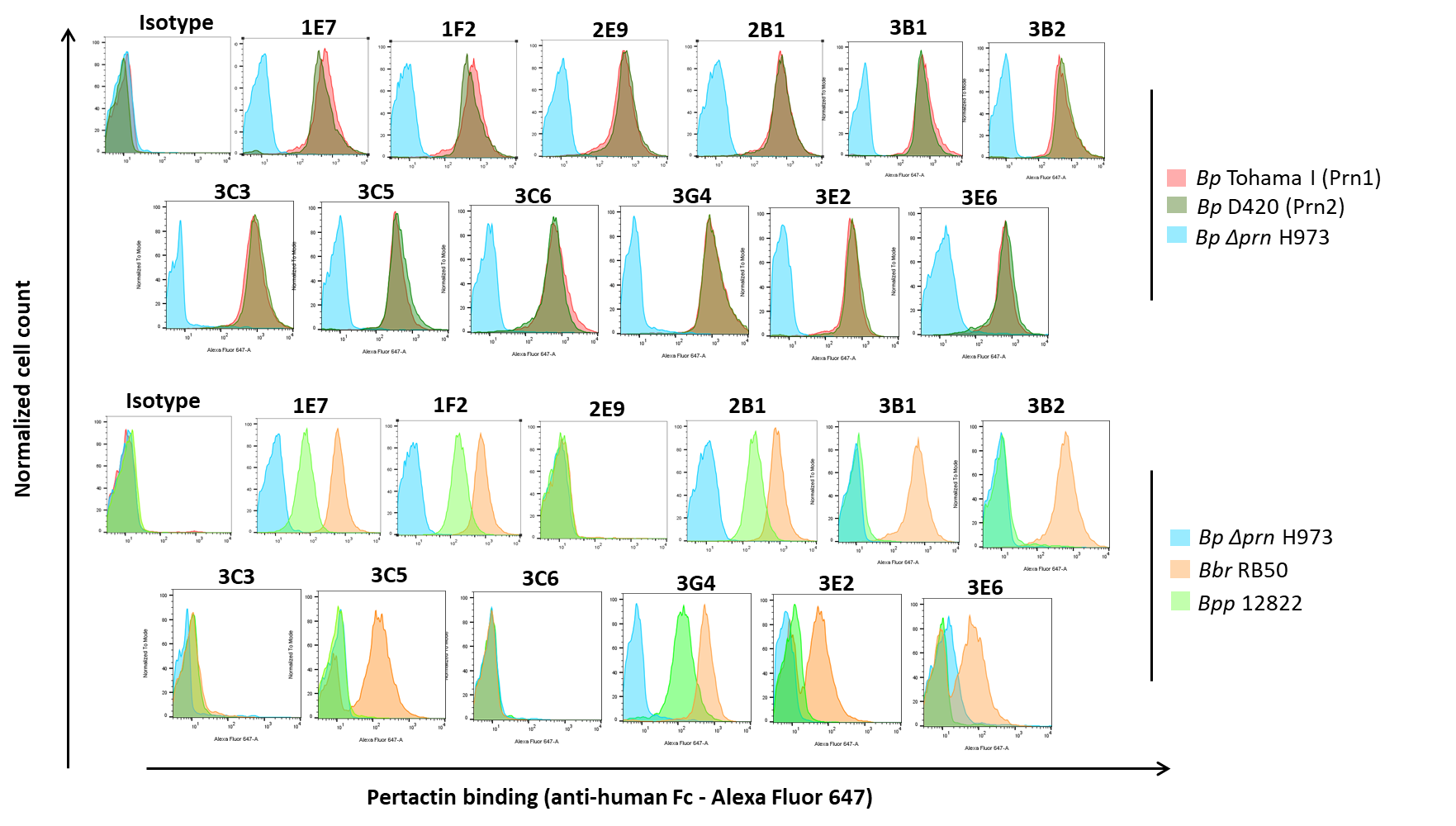


**Figure S4. Antibodies bind cell-associated pertactin.** Log-phase *B. pertussis* TohamaI (expressing Prn1) and D420 (expressing Prn2), the pertactin-deficient strain H973, *B. bronchiseptica* RB50 and *B. parapertussis* 12822 were stained with 20 ug/ml anti-pertactin IgG1 or an isotype control antibody, followed by goat anti-human Fc-AF647 secondary antibody and assessed for antibody binding using Fortessa flow cytometer. *Bp, B. pertussis; Bbr, B. bronchiseptica; Bpp, B. parapertussis.*


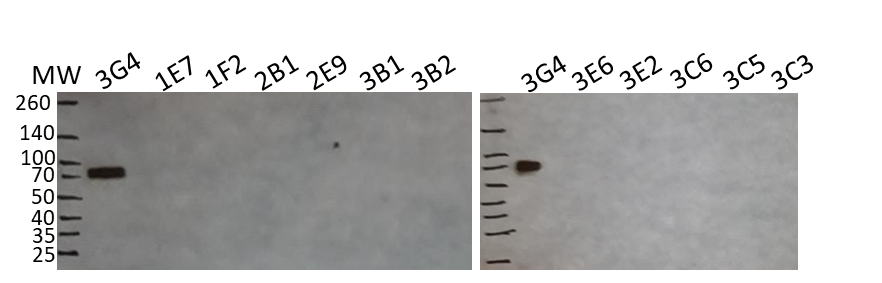


**Figure S5. Only antibody 3G4 detects pertactin on a Western blot.** Pertactin (Prn1, 100 ng) was separated by 4-20% gradient SDS-PAGE gel, transferred to PVDF membrane and each lane probed separately with the indicated antibody at 0.5 µg/ml followed by detection with anti-human IgG Fc-HRP and chemiluminescent substrate.


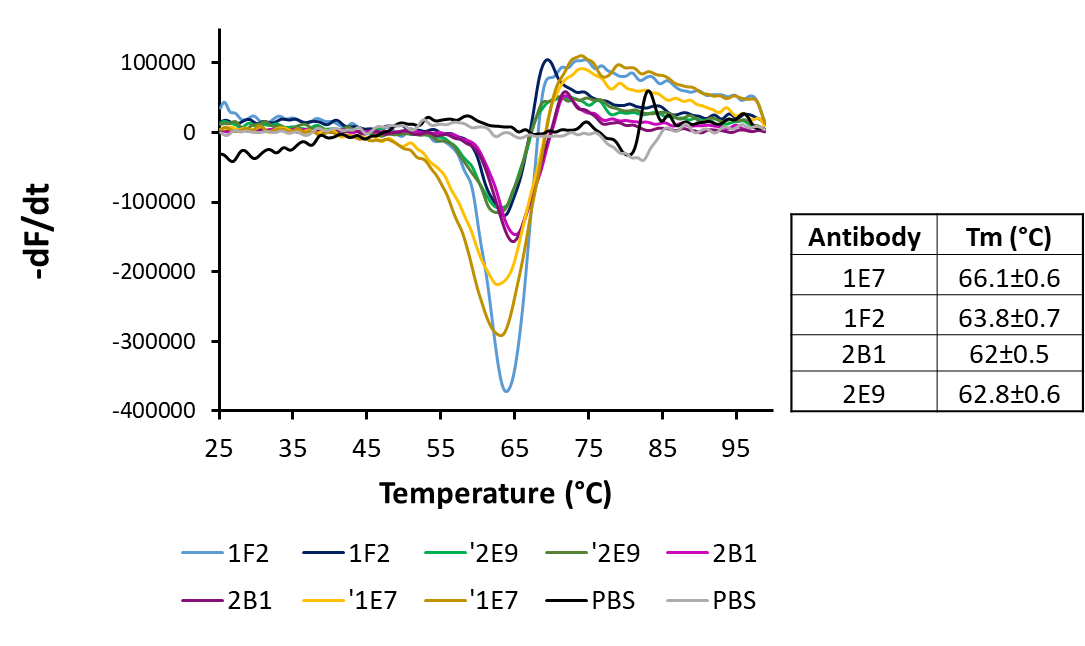


**Figure S6. Antibody thermal unfolding.** The thermal unfolding temperatures of the four lead antibodies were assessed in duplicates using the Protein Thermal Shift Dye Kit (ThermoFisher Scientific). The Fab thermal unfolding temperatures of the four lead antibodies as mouse IgG2a were assessed in duplicates using the Protein Thermal Shift Dye Kit. Average and standard deviation reported.

**Figure S7.** Weight changes from lethal mouse challenge in Fig. 6B Weights of each mouse was recorded daily. Mouse weight averages of group receiving isotype or prn cocktail reported. No mouse receiving an isotype survived past day 2.


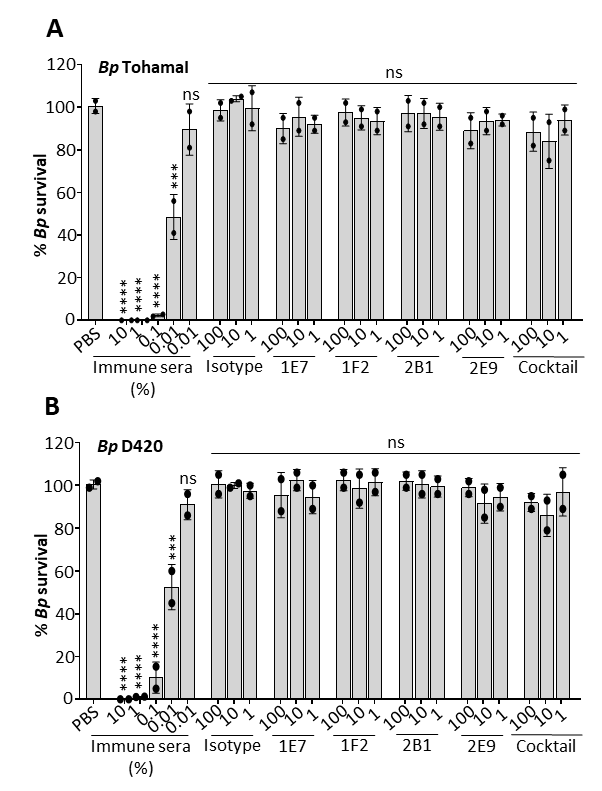


**Figure S8. Antibody-meditated complement dependent lysis of *Bp*** Antibody-mediated complement killing as in Fig. 6, here showing results with 1E7, 1F2, 2B1 and 2E9 individually for **A,** TohamaI and **B,** D420. The results were normalized with the number of surviving bacteria recovered after incubation with only naïve sera set at 100%. Results reported show average of two independent experiments, with two technical replicates each.
